# New frontiers in CRISPR: Addressing antimicrobial resistance with Cas9, Cas12, Cas13, and Cas14

**DOI:** 10.1016/j.heliyon.2025.e42013

**Published:** 2025-01-18

**Authors:** Ahmed S.A. Ali Agha, Ali Al-Samydai, Talal Aburjai

**Affiliations:** a School of Pharmacy, Department of Pharmaceutical Sciences, The University of Jordan, Amman, 11942, Jordan; b Pharmacological and Diagnostic Research Center, Department of Pharmaceutical Sciences, Faculty of Pharmacy, Al-Ahliyya Amman University, (AA), Amman, 19328, Jordan

**Keywords:** CRISPR-Cas, Superbugs, Anti-microbial resistance, Gene-editing, Antimicrobial therapy, Cas12, Cas13, Cas14

## Abstract

**Background:**

The issue of antimicrobial resistance (AMR) poses a major challenge to global health, evidenced by alarming mortality predictions and the diminishing efficiency of conventional antimicrobial drugs. The CRISPR-Cas system has proven to be a powerful tool in addressing this challenge. It originated from bacterial adaptive immune mechanisms and has gained significant recognition in the scientific community.

**Objectives:**

This review aims to explore the applications of CRISPR-Cas technologies in combating AMR, evaluating their effectiveness, challenges, and potential for integration into current antimicrobial strategies.

**Methods:**

We conducted a comprehensive review of recent literature from databases such as PubMed and Web of Science, focusing on studies that employ CRISPR-Cas technologies against AMR.

**Conclusions:**

CRISPR-Cas technologies offer a transformative approach to combat AMR, with potential applications that extend beyond traditional antimicrobial strategies. Integrating these technologies with existing methods could significantly enhance our ability to manage and potentially reverse the growing problem of antimicrobial resistance. Future research should address technical and ethical barriers to facilitate safe and effective clinical and environmental applications. This review underscores the necessity for interdisciplinary collaboration and international cooperation to harness the full potential of CRISPR-Cas technologies in the fight against superbugs.

## 1 Introduction

### 1.1 The rising crisis of antimicrobial resistance and the urgent need for innovative solutions

The Nevada tragedy of 2016, when an untreated bacterial infection claimed a woman's life, highlighted antimicrobial resistance (AMR), a dangerous global health issue [1]. This case revealed the risks posed by AMR and highlighted its urgent need for attention. The event also demonstrated how quickly such an illness can spread, highlighting the importance of addressing this major public health significance. This life-threatening phenomenon, accelerated by the overuse of antibiotics and the evolution of multidrug-resistant "superbugs," is linked to roughly 700,000 deaths yearly [2]. Without intervention, this number could skyrocket to a staggering 10 million deaths by 2050 - surpassing cancer as humanity's leading cause of mortality [3,4]. Unfortunately, progress in new drugs has stalled, leaving healthcare professionals with few solutions for resistant diseases. International health organizations now demand inventive approaches; luckily, CRISPR-Cas technology provides hope. This precise and flexible gene-editing tool offers numerous potential applications, such as novel treatments against infections, diagnostics development, and fresh antimicrobial strategies – presenting us with an unprecedented opportunity to combat AMR effectively [5].

Given the increasing global threat of AMR, the effectiveness of traditional antibiotics is diminishing. This review comprehensively analyzes recent developments, their impact, and their challenges. It aims to guide future research and applications in addressing AMR.

### 1.2 Historical perspective

The ongoing battle against superbugs has primarily relied on conventional approaches such as the discovery of novel substances or modifications to current medications. However, it is frequently necessary to reassess these endeavors [6]. The emergence of CRISPR-Cas technology, a gene-editing technique derived from natural bacterial defense systems, represents unprecedented specificity, adaptability, and ease of use and notable progress in this ongoing battle [[7], [8], [9]] as illustrated in Table 1.

**Table 1:** CRISPR-Cas strategies against antimicrobial resistance, summarizing key applications, advantages, challenges, and future potential.

Therapeutic Applications				
Application	CRISPR-Cas Strategy	Advantages	Challenges	References
Targeted antimicrobial therapy	Design CRISPR-Cas systems to selectively target and disable resistance-conferring genes or induce bacterial cell death	High specificity, minimal impact on host microbiota	Potential off-target effects, delivery challenges	[19]
Bacterial genome engineering	Use CRISPR-Cas to create engineered bacterial strains with reduced pathogenicity or susceptibility to antimicrobials	Potential for studying virulence factors and resistance mechanisms	Ethical concerns, unintended consequences	[20]
Enhancing host immunity	Utilize CRISPR-Cas to modify host immune cells, increasing their ability to recognize and eliminate antibiotic-resistant bacteria	Boosts host defenses, potential for personalized therapy	Ethical concerns, safety, and delivery challenges	[21]
Bacterial persistence targeting	Target and eliminate bacterial persister cells, which can survive antibiotic treatment and repopulate infections, using CRISPR-Cas systems	Addresses a key factor in recurrent infections	Delivery challenges, potential off-target effects	[22]
Novel antimicrobial agent development	Use CRISPR-Cas to create modified antimicrobial peptides or other novel agents, bypassing existing resistance mechanisms	Opportunity for innovative, highly effective antimicrobial agents	Cost, complexity of development, regulatory approval process	[23]
Anti-virulence strategies	Leverage CRISPR-Cas to target and disrupt bacterial virulence factors, reducing pathogenicity without exerting selective pressure for resistance development	Minimizes selective pressure, potential for combination therapies	Delivery challenges, potential off-target effects	[24]
Phage therapy enhancement	Employ CRISPR-Cas to modify bacteriophages, increasing their efficacy against resistant bacteria or enabling them to target specific bacterial strains	Potential for highly specific, effective antimicrobial therapy	Phage resistance, regulatory approval process	[25]
CRISPR-Cas-based vaccines	Develop CRISPR-engineered bacterial strains that can serve as live vaccines, stimulating the immune system without causing disease	Potential for long-lasting, broad-spectrum protection	Safety concerns, regulatory approval process	[26]
Prophylactic CRISPR-Cas systems	Introduce CRISPR-Cas systems into susceptible hosts or microbiota, providing resistance against incoming pathogens	Prevention of infections, potential for reducing AMR spread	Delivery challenges, ecological considerations	[27]
Diagnostic Applications				
Rapid diagnostics	Use CRISPR-Cas tools to detect specific genetic signatures of resistant bacteria, enabling rapid identification of resistance profiles	Speed, accuracy, potential for point-of-care diagnostics	Development of robust, user-friendly diagnostic platforms	[28]
Synthetic biology and biosensors	Develop CRISPR-Cas-based biosensors to detect and respond to the presence of antimicrobial-resistant bacteria, triggering tailored responses	Real-time detection and response, customizable	Complexity of design, implementation challenges	[29]
Environmental Applications				
Environmental applications	Utilize CRISPR-Cas to reduce the spread of resistance genes in agricultural and aquacultural settings, as well as in wastewater treatment	Potential for large-scale impact on AMR spread	Ethical and ecological concerns, regulatory approval process	[30]
Combating resistance gene transfer	Use CRISPR-Cas to disrupt plasmids, transposons, or other mobile genetic elements responsible for resistance gene transfer among bacteria	Reduces horizontal gene transfer, limits AMR spread	Delivery challenges, potential off-target effects	[31]
Other Applications				
Quorum sensing interference	Employ CRISPR-Cas to disrupt quorum sensing, the communication system used by bacteria to coordinate behaviors, including resistance mechanisms	Interferes with bacterial coordination and resistance	Delivery challenges, potential off-target effects	[32]
Biofilm disruption	Use CRISPR-Cas to target and disrupt genes involved in biofilm formation, rendering bacteria more susceptible to antimicrobials	Enhances antimicrobial efficacy, reduces chronic infections	Delivery challenges, potential off-target effects	[33]
Resistance mechanism investigation	Employ CRISPR-Cas-mediated knockout screens to identify genes involved in antibiotic resistance, providing insights into the molecular basis of drug resistance	Systematic, high-throughput investigation of gene function	Time-consuming, labor-intensive	[34]
CRISPR-Cas interference (CRISPRi)	Use deactivated Cas9 (dCas9) to block transcription of resistance genes or essential bacterial genes, inhibiting bacterial growth and resistance	Precise, reversible gene regulation	Delivery challenges, potential off-target effects	[35]

**Footnote:** CRISPR (Clustered Regularly Interspaced Short Palindromic Repeats), AMR (Antimicrobial Resistance), dCas9 (deactivated Cas9).

**Fig. 1:**
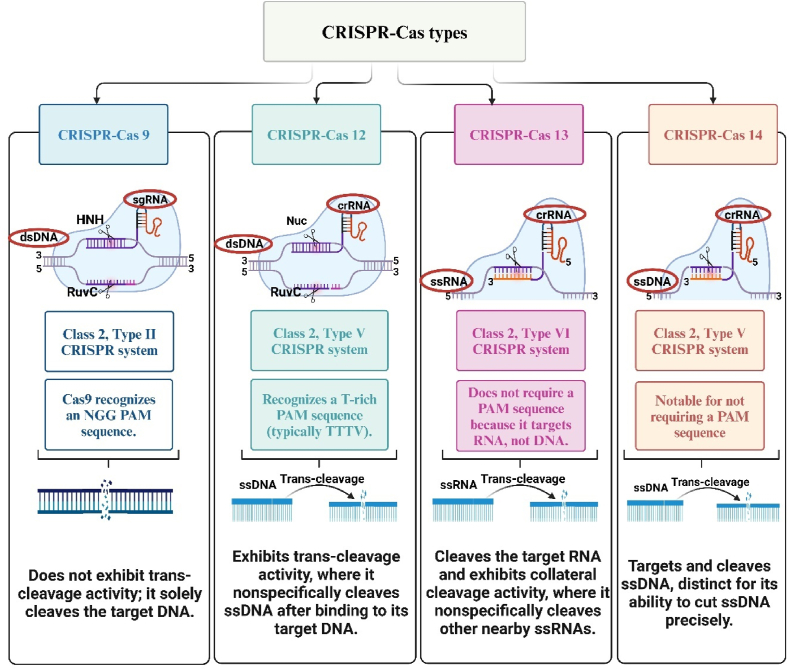
Overview of CRISPR-Cas system types, highlighting the mechanisms, target specificities, and trans-cleavage activities of Cas9, Cas12, Cas13, and Cas14.

CRISPR-Cas systems, including Cas9, Cas12, Cas13, and the recently identified Cas14, offer precise, adaptable, and promising tools for combating antimicrobial resistance [10]. Fig. 1 provides an overview of these CRISPR-Cas systems, highlighting their key characteristics and applications.

Cas9 is the most extensively studied and utilized, known for its ability to introduce targeted double-strand breaks in DNA guided by a single guide RNA (sgRNA) [11]. Cas12, particularly Cas12a, shares similar DNA-targeting capabilities but also provides the unique ability to cleave single-stranded DNA (ssDNA) nonspecifically after activation, a feature that enhances its utility in diagnostic applications [12]. Cas13 distinguishes itself by targeting RNA rather than DNA, making it a powerful tool for RNA-based diagnostics and therapies [13]. Cas14, notable for its small size, can target ssDNA with high specificity without requiring a PAM sequence, broadening its potential applications in precise genome editing [14].

These CRISPR-Cas systems are not only advancing targeted therapies that selectively eliminate resistant bacteria while preserving beneficial microbiota [[15], [16], [17]]. As a research tool for illuminating molecular mechanisms behind antimicrobial resistance and potential intervention targets for the future, CRISPR-Cas is changing the game in combatting this global crisis [18]. However, it has limitations; off-target effects must be closely monitored to ensure safe application alongside ethical considerations made through interdisciplinary collaboration.

## 2 Exploiting CRISPR-cas capabilities for neutralizing antibiotic-resistant bacteria

CRISPR-Cas technology has enabled the development of precise antibacterial strategies due to its extraordinary specificity and adaptability [36]. By customizing guide RNA molecules, CRISPR-Cas systems can target unique genetic sequences in resistant bacteria [37], leading to innovative applications such as disabling resistance genes in *Klebsiella pneumoniae* [38]. Gene disruption is a viable approach for utilizing CRISPR-Cas technology against antibiotic resistance by directly targeting and deactivating the responsible genes [39]. Studies have successfully illustrated this technique in *Staphylococcus aureus* [40], rendering it susceptible once more to antibiotics after gene inhibition. Additionally, sequence-specific bacterial erosion through CRISPR antimicrobial nucleases (CANs) effectively eradicates tough bacteria without disturbing beneficial microbiota via targeted DNA fragmentation [41]. The ongoing development of novel CRISPR variants, including Cas13 and base/prime editors, progressively broadens the scope for antibacterial applications with this transformative technology [42].

## 3 Exploiting bacterial mechanisms: leveraging the natural origins of CRISPR-Cas for antimicrobial applications

CRISPR-Cas systems have revolutionized bacterial defense, providing a robust natural adaptive immune response for bacteria and archaea [43]. This cutting-edge gene editing technology has been effectively utilized to combat antimicrobial resistance [44], using these precise techniques as an alternative to traditional antibiotics. To enhance bacteriophage therapy, researchers are leveraging CRISPR-Cas9 technology by engineering phages to deliver this system into bacterial cells, which precisely targets and disrupts drug resistance genes [45]. This approach selectively eradicates resistant bacteria while sparing beneficial microbes [45]. Additionally, these engineered phages can carry genetic payloads, such as biofilm-degrading enzymes, to further improve antimicrobial efficacy, representing a significant advancement in combating antibiotic-resistant infections [45]. Moreover, scientists can exploit bacterial quorum sensing by interfering with its communication pathways through CRISPR-Cas systems; targeting related genes weakens populations of bacteria, making them susceptible to existing antibiotics – offering potential combination therapies that could increase efficacy overall. Bikard and Barrangou (2017) discuss how various CRISPR-Cas systems, particularly Type I and II, can be engineered to selectively eliminate pathogenic bacteria by targeting and cleaving specific chromosomal sequences. Delivered through phages or phagemids, these systems have shown the ability to efficiently eradicate resistant bacteria or eliminate plasmids carrying antibiotic-resistance genes. This approach not only targets pathogens with high specificity but also minimizes the impact on beneficial microbiota, offering a promising alternative to traditional broad-spectrum antibiotics [46].

Earlier, Bikard et al. (2014) developed a CRISPR-Cas9-based antimicrobial approach that selectively targets and eliminates virulent strains of *Staphylococcus aureus* by destroying antibiotic resistance genes. This sequence-specific targeting was effective in vivo, as demonstrated in a mouse skin colonization model, highlighting the potential of CRISPR-Cas9 as a powerful tool against multidrug-resistant pathogens [47].

CRISPR-Cas is a versatile tool that can effectively address even the most challenging microbial issues, as shown in Table 2. They can be used in conjunction with other strategies, such as capitalizing on bacterial competition and microbiota modulation, unleashing the power of CRISPR-Cas systems [48] enables researchers to promote the growth of beneficial bacteria while suppressing the expansion of pathogens via targeted elimination of specific strains from their rivals - giving us unprecedented control over therapeutic agents without side effects like those associated with traditional treatments like antibiotics. As research advances, CRISPR-Cas holds substantial promise in addressing global antimicrobial resistance, necessitating careful consideration of its scientific impact and ethical issues, including ecological effects and Genetically Modified Organism (GMO) utilization.

**Table 2:** CRISPR-Cas systems against bacterial antimicrobial resistance, detailing targeted genes, mechanisms, and advantages in combating resistant infections.

Bacterial Species	CRISPR-Cas System	Target Resistance Gene(s)	Mechanism of Action	Application Area	Key Advantages	Reference
*Streptococcus pyogenes*	Cas9	*mecA, blaZ*	Cleavage of resistance genes in target bacteria	Treatment of MRSA infections	High specificity, reduced off-target effects	[49]
*Francisella novicida*	Cas9	*FPI* genes	Inhibition of intracellular growth and virulence	Attenuation of pathogen virulence	Potential vaccine development, improved understanding of virulence	[50]
*Campylobacter jejuni*	Cas9	*blaOXA-61*	Cleavage of resistance gene, reduction in β-lactam resistance	Treatment of *Campylobacter* infections	Improved antibiotic susceptibility, potential for reduced resistance development	[51]
*Streptococcus thermophilus*	Cas9	*ermB,*	Targeting and destruction of antibiotic-resistant plasmids	Treatment of *Streptococcus* infections	Prevention of horizontal gene transfer, reduced resistance	[52]
*Klebsiella pneumoniae*	Cas3	*blaKPC*	Selective elimination of carbapenem-resistant strains	Treatment of carbapenem-resistant *K. pneumoniae* infections	High specificity, reduced off-target effects, potential for combinatorial therapy	[21]
*Escherichia coli*	Cas3	*blaNDM-1*	Disruption of resistance genes, selective elimination of target bacteria	Treatment of multidrug-resistant *E. coli* infections	High specificity, efficient removal of resistant bacteria	[53]
*Pseudomonas aeruginosa*	Cas12a	*mexZ*	Targeting and cleavage of multidrug efflux pump genes	Treatment of *P. aeruginosa* infections	Improved antibiotic susceptibility, reduced resistance	[54]
*Enterococcus faecalis*	Cas9	*vanA, vanB*	Targeting and destruction of vancomycin resistance genes	Treatment of *VRE* infections	High specificity, restoration of vancomycin sensitivity	[55]
*Neisseria meningitidis*	Cas9	*penA*	Disruption of penicillin resistance gene, restoration of susceptibility	Treatment of *N. meningitidis* infections	Enhanced antibiotic efficacy, potential for combinatorial therapy	[56]
*Acinetobacter baumannii*	Cas12a	*blaOXA-23*	Targeting and cleavage of carbapenem resistance gene	Treatment of carbapenem-resistant *A. baumannii* infections	High specificity, potential for combinatorial therapy	[54]
*Mycobacterium tuberculosis*	Cas9	*rpoB, katG*	Disruption of rifampicin and isoniazid resistance genes	Treatment of multidrug-resistant tuberculosis	Enhanced treatment efficacy, potential for personalized therapy	[57]
*Lactobacillus plantarum*	Cas9	Various	Use of bacteriocin-producing probiotic bacteria armed with CRISPR-Cas9	Probiotic therapy for gut infections	Enhanced antimicrobial activity, improved gut health	[58]

**Footnote:** MRSA (Methicillin-Resistant Staphylococcus aureus), FPI (Francisella Pathogenicity Island), blaZ (β-lactamase gene), ermB (erythromycin resistance gene), blaKPC (Klebsiella pneumoniae carbapenemase), blaNDM-1 (New Delhi metallo-β-lactamase 1), mexZ (multidrug efflux pump repressor gene), vanA and vanB (vancomycin resistance genes), penA (penicillin-binding protein gene), blaOXA-23 (oxacillinase gene), rpoB (RNA polymerase beta subunit gene), katG (catalase-peroxidase gene).

## 4 Revolutionizing diagnostics: CRISPR-Cas tools for rapid detection of antimicrobial resistance

The increasing problem of antimicrobial resistance (AMR) has prompted the development of efficient and precise diagnostic methods to identify and categorize resistant pathogens. CRISPR-Cas systems such as Cas9, Cas12a, and Cas13 have been utilized to create a variety of inventive diagnostics that specifically target resistance genes in an array of microbes [38]. These technologies boast multiple benefits when compared with standard diagnosis techniques, including swift analysis timeframes, heightened accuracy, and potential trial for portability and ease of use [38]. Several CRISPR-Cas-based tools have been developed to identify resistant bacteria strains as mentioned in Table 3 - SHERLOCK, DETECTOR, or the Cas13a assay are employed for detection in *E. coli*, *S. aureus* & *K. pneumonia*, respectively [38,59,60]. Similarly, other tests like those based on Cas12a & 13 can be used to swiftly spot genetic markers associated with drug resistance in *N. gonorrhea, M. tuberculosis & P. aeruginosa* [[61], [62], [63]]. Different analytical methodologies may be adopted depending on need – lateral flow strips, fluorometric testing, real-time fluorescence readouts, or electrochemical reactions are some examples, amongst many more available options offering flexibility while choosing a test type. Recent developments in this field have expanded the scope by offering specialized diagnostic measures towards *Enterococcus species, Salmonella enterica, Staphylococcus epidermidis*, etc. [[64], [65], [66], [67], [68]].

**Table 3:** CRISPR-Cas diagnostics for antimicrobial resistance, detailing methods, targets, and key advantages in rapid and precise detection.

Diagnostic Tool	Target Pathogen	Target Resistance Gene(s)	Detection Method	Key Advantages	Reference
CRISPR-Chip	*Staphylococcus epidermidis*	*mecA*	Electrical readout	Label-free, sensitive, high specificity, low sample preparation	[64]
DETECTR	*Staphylococcus aureus*	*mecA*	Fluorometric detection	High specificity, quantitative analysis, low-cost	[60]
CRISPR-Cas12a assay	*Neisseria gonorrhoeae*	*23S rRNA*	Lateral flow strip	Rapid, point-of-care diagnosis, specific, and sensitive	[63]
Cas13-based RT-LAMP	*Mycobacterium tuberculosis*	*rpoB*	Real-time fluorescence	Simultaneous detection of multiple resistance genes, high-throughput	[62]
Cas9-based PCR-free assay	*Pseudomonas aeruginosa*	*bla_OXA, bla_VIM, bla_IMP*	Fluorescence readout	PCR-free, rapid, sensitive, and specific	[61]
Cas13a-based assay	*Klebsiella pneumoniae*	*bla_KPC*	Fluorescence readout	High sensitivity, adaptable to various resistance genes	[59]
Cas13-based electrochemical detection	*Escherichia coli*	*bla_TEM, bla_SHV, bla_CTX-M*	Electrochemical readout	Label-free, portable, sensitive, and specific	[67]
Cas12a-based paper-based assay	*Salmonella enterica*	*qnrS, bla_CTX-M*	Colorimetric detection	Rapid, low-cost, portable, easy-to-use	[69]
Cas12a-based portable smartphone detection	*Campylobacter jejuni*	*tet(O)*	Smartphone readout	Rapid, portable, easy-to-use, low-cost	[68]
SHERLOCK	*Escherichia coli*	*bla_CTX-M*	Lateral flow strip	Rapid, sensitive, specific, easy-to-use	[38]
Cas12a-based multiplex detection	*Enterococcus species*	*vanA, vanB*	Lateral flow strip	Simultaneous detection of multiple resistance genes, rapid, easy-to-use	[65]

**Footnote:** CRISPR (Clustered Regularly Interspaced Short Palindromic Repeats), RT-LAMP (Reverse Transcription Loop-Mediated Isothermal Amplification), PCR (Polymerase Chain Reaction), SHERLOCK (Specific High-sensitivity Enzymatic Reporter unLOCKing), mecA (methicillin resistance gene), bla (β-lactamase gene), rpoB (RNA polymerase beta subunit gene), 23S rRNA (23S ribosomal RNA), tet(O) (tetracycline resistance gene).

### 4.1 Innovations in biosensing: the role of CRISPR-Cas systems in modern diagnostics

Integrating CRISPR/Cas systems into biosensing technologies significantly advances the rapid, specific, and sensitive detection of pathogenic bacteria and viruses [12]. This emerging field combines the precision of CRISPR-based gene editing with the versatility of biosensors, offering new solutions for food safety, clinical diagnostics, and environmental monitoring.

### 4.2 Nanomaterials-assisted CRISPR/Cas detection

One of the promising developments in this area is using nanomaterials to enhance CRISPR/Cas-based detection platforms. These nanomaterials can improve the sensitivity and specificity of CRISPR-based biosensors by facilitating the immobilization of CRISPR components or enhancing signal transduction [70]. The conducted review by Zhao et al. (2023) on nanomaterials-assisted CRISPR/Cas detection highlights the potential of nanomaterials-assisted CRISPR/Cas detection systems in enhancing food safety [71]. The integration of nanomaterials, such as quantum dots, gold nanoparticles, and carbon nanomaterials, with CRISPR/Cas biosensors significantly improves detection sensitivity, specificity, and versatility across various food safety applications [71], including identifying foodborne pathogens, toxins, and genetically modified organisms [71].

Also, a study by Peng et al. developed a novel nano-sieve device integrated with CRISPR/Cas technology to detect methicillin-resistant *Staphylococcus aureus* (MRSA) [72]. The device uses a pneumatically-regulated chamber and magnetic beads to concentrate bacterial cells, achieving a 15-fold concentration factor and a limit of detection (LOD) of approximately 100 CFU/mL [72]. This approach significantly enhances the sensitivity and efficiency of CRISPR-based biosensing, offering a rapid and effective solution for point-of-care diagnostics in resource-limited settings.

### 4.3 CRISPR/Cas systems beyond nucleic acids

Traditionally, CRISPR-Cas systems have been employed for detecting nucleic acids, but recent advancements have expanded their use to non-nucleic acid targets [73]. This diversification is driven by the system's adaptability and the development of novel CRISPR/Cas-based biosensors capable of detecting proteins, small molecules, and other analytes [74]. The conducted study by Quan et al. developed the FLASH (Finding Low Abundance Sequences by Hybridization) system to multiplex the detection of AMR genes [75]. By utilizing CRISPR/Cas9 technology, FLASH enhances the enrichment of target DNA sequences by up to five orders of magnitude, allowing for the identification of AMR genes even at sub-attomolar concentrations [75]. This system enables the simultaneous detection of multiple resistance markers in clinical samples, such as respiratory fluids and dried blood spots, making it a valuable tool for managing multidrug-resistant infections [75]. Also, the study by Suea-Ngam et al. introduced an innovative amplification-free CRISPR/Cas12a-based electrochemical biosensor specifically designed to detect MRSA [60]. This system employs a custom-designed guide RNA targeting the *mecA* gene of MRSA, combined with silver metallization, to achieve highly sensitive detection. The biosensor demonstrated an impressive detection limit of 3.5 fM, and was successfully applied in human serum, positioning it as a promising tool for field-deployable diagnostics in managing antimicrobial resistance [60].

### 4.4 Ultrasensitive detection of pathogens

Combining CRISPR/Cas systems with advanced fluorescent biosensing technologies has created highly sensitive platforms capable of detecting pathogens at very low concentrations.

For instance, the study conducted by Li et al. developed an electrochemical biosensor using CRISPR/Cas12a to detect Listeria monocytogenes, a pathogen associated with foodborne illnesses and resistance [76]. The biosensor displayed an LOD of 26 CFU/mL and proved effective in both spiked and natural food samples, showcasing its potential for rapid and sensitive detection of AMR pathogens in real-world settings [76].

Another study conducted by Wei et al. introduced a CRISPR/Cas12a-based magnetic relaxation switching (C-MRS) biosensor for the amplification-free detection of MRSA [29]. The biosensor achieved an LOD of 16 CFU/mL in food samples, demonstrating high sensitivity and accuracy without the need for nucleic acid pre-amplification, which is crucial for reducing false positives in AMR detection [29].

### 4.5 Isothermal amplification and CRISPR/Cas integration

Another promising approach involves integrating isothermal amplification techniques with CRISPR/Cas systems. This combination allows for the rapid and amplification-free detection of nucleic acids, simplifying the diagnostic process and reducing the need for complex laboratory equipment. For instance, the conducted study by Cao et al. developed a CRISPR/Cas12a-based platform integrated with loop-mediated isothermal amplification (LAMP) to detect MRSA rapidly [65]. The system demonstrated a high sensitivity with a limit of detection (LOD) of 1 aM (∼1 copy μL−1) and showed 100 % specificity and sensitivity in clinical bacterial isolates within approximately 80 min [65]. This integration allows for effective point-of-care diagnostics in identifying AMR pathogens. Also, Xu et al. developed a method combining loop-mediated isothermal amplification (LAMP) with CRISPR/Cas12a and a lateral flow immunochromatographic strip to detect carbapenem-resistant *Klebsiella pneumoniae* and *New Delhi metallo-β-lactamase* (NDM) genes [77]. This assay detects bacteria directly in samples with a concentration as low as 3 × 105 CFU/mL without bacterial culture, offering a rapid and sensitive solution for identifying resistant strains in clinical settings [77].

## 5 Precision medicine and CRISPR-Cas: an integrated strategy for addressing antimicrobial resistance

The integration of precision medicine and CRISPR-Cas technology can revolutionize antimicrobial therapy in the fight against growing microbial resistance [38]. Addressing a tailored approach, precision medicine allows us to target resistant pathogens while preserving essential elements of the host's microbiome [25]. Using sequence-specific targeting, CRISPR-Cas systems can be a versatile platform for developing such therapies, selectively eliminating resilient bacteria, and reducing the risk of dysbiosis [24]. One promising strategy is utilizing bacteriophages with these systems to create highly specific antimicrobial agents that eradicate resistant microbes without exhibiting any drawbacks associated with traditional antibiotics [78]; an example is successful treatment using personalized phage cocktails for multidrug-resistant *Acinetobacter baumannii* infections [79]. Furthermore, gene editing through CRISPR-Cas9 may also be employed by disabling resistance genes on microorganisms like MRSA (methicillin-resistant *Staphylococcus aureus*) [52], this increases their susceptibility to current antibiotics, enhancing their effectiveness and reducing the demand for new drug research and development.

## 6 Interdisciplinary collaboration: advancing CRISPR-Cas solutions against antimicrobial resistance

Organizations like IGI and IPATH exemplify the benefits of multi-disciplinary collaboration, merging basic and applied research to innovate solutions. Computational tools such as CRISPR-offender enhance gene editing safety and facilitate translational medicine by predicting off-target effects [80]. In bioengineering, developing delivery systems like lipid-polymer hybrid nanoparticles is crucial for the clinical application of CRISPR Cas9 therapies as it has great potential for improving the delivery efficiency and stability of CRISPR/Cas9. These hybrid systems can provide better control over particle size, loading capacity, and release profiles, making them suitable for various therapeutic applications [81]. Educational initiatives, such as “CRISPR in the Classroom,” are critical in training researchers to appreciate the importance of interdisciplinary collaboration [82]. Moreover, global partnerships, like the “CRISPR for Health” consortium, leverage open science to accelerate development by integrating worldwide scientific expertise [83].

## 7 CRISPR-Cas applications in agriculture, aquaculture, and biocontrol: advancing sustainability and productivity

The CRISPR-Cas system is a revolutionary tool that enables the modification of organisms across various industries, such as agriculture, aquaculture, and biocontrol [84]. This technology provides numerous advantages over traditional methods through gene editing to boost resistance or immunity. Regarding crop production, it has been used to create wheat and rice varieties with enhanced tolerance against diseases like *Lr34* and *Xa21*, respectively [85,86]. Consequently, farmers can reduce their reliance on chemical pesticides and adopt more sustainable farming practices. Also, in livestock breeding, animals have been given increased protection from porcine reproductive respiratory syndrome (PRRS) via targeting the *CD163* gene [87], along with bovine spongiform encephalopathy (BSE) by focusing on the *PRNP* gene [88]- ultimately leading to better animal health and productivity. In addition, bacterial biocontrol agents using CRISPR-Cas systems like Cas9 and Cas12, which effectively target pathogens while editing genes related to resistance, e.g., *cA*, *blaNDM-1*, thus providing an environmentally friendly alternative chemical pesticide usage, contributing towards sustainable pest management strategies. In aquaculture, the CRISPR-Cas technology has been employed to enhance the resilience and disease resistance of various strains, improving their health and productivity. For instance, *tlr5* gene editing was implemented on tilapia [89], and *saCas9* gene editing on Atlantic salmon [90]. Additionally, *RPS3* and *MyD88* genes were targeted in Pacific oysters for enhanced immune response [91], while shrimp resistance against white spot syndrome virus (WSSV) was increased by modifying the *WSSV* gene itself [92]. This breakthrough of biotechnology can even be utilized further to engineer probiotic bacteria capable of combating pathogens such as *Streptococcus* and *Vibrio cholerae* through targeting respective genes like *LytA*, *ctxA*, and *ctxB* [93,94] leading towards a better aquatic environment with improved water quality - consequently promoting sustainable aquacultural practices. CRISPR-Cas applications have enhanced production efficiencies while concurrently promoting environmental conservation, as illustrated in Table 4; it is therefore clear that this technology has significantly contributed to agriculture/aquaculture advancement.

**Table 4:** CRISPR-Cas in agriculture and aquaculture, targeting antimicrobial resistance, boosting productivity, and promoting sustainability.

Application	Organism	Target Resistance Gene(s)	CRISPR-Cas System Used	Outcome of Modification	Key Advantages	Reference
Agriculture	Wheat	*Lr34*	Cas9	Disease-resistant crop varieties	Reduced need for chemical pesticides	[85]
	Rice	*Xa21*	Cas9	Disease-resistant crop varieties	Reduced need for chemical pesticides	[86]
	Pigs	*CD163*	Cas9	PRRS-resistant pigs	Improved animal health and productivity	[87]
	Bacterial biocontrol agents	*MecA, blaNDM-1*	Cas9, Cas12a	Targeted killing of pathogens	Eco-friendly alternative to chemical pesticides	[95]
	Cattle	*PRNP*	Cas9	BSE-resistant cattle	Improved animal health and productivity	[88]
Aquaculture	Probiotic bacteria	*ctxA, ctxB*	Cas9	Lysis of *V. cholerae*	Improved water quality and fish health	[93]
	Probiotic bacteria	*LytA*	Cas9, Cas12a	Lysis of *Streptococcus*	Improved water quality and fish health	[94]
	Atlantic salmon	*saCas9*	Cas9	Disease-resistant fish strains	Improved fish health and productivity	[90]
	Pacific oyster	*MyD88*	Cas9	Enhanced immune response	Increased yield and reduced disease outbreaks	[91]
	Shrimp	*WSSV*	Cas9	Enhanced resistance to WSSV	Increased yield and reduced disease outbreaks	[92]
	Tilapia	*tlr5*	Cas9	Disease-resistant fish strains	Improved fish health and productivity	[89]

**Footnote:** Lr34 (Leaf rust resistance gene 34), Xa21 (bacterial blight resistance gene), CD163 (cluster of differentiation 163), PRRS (Porcine Reproductive and Respiratory Syndrome), PRNP (prion protein gene), BSE (Bovine Spongiform Encephalopathy), mecA (methicillin resistance gene), blaNDM-1 (New Delhi metallo-β-lactamase 1), ctxA and ctxB (cholera toxin genes), LytA (autolysin gene), MyD88 (myeloid differentiation primary response 88 gene), WSSV (White Spot Syndrome Virus), tlr5 (toll-like receptor 5 gene).

## 8 Challenges and opportunities

Technical issues such as off-target effects and unintended consequences can be addressed through improved specificity, CRISPR-Cas systems design [96], and enhanced delivery methods like nanoparticles [97]. To attenuate the emergence of resistance to these therapies, combinatorial treatments should be utilized alongside consistent updating of targeting sequences [98]. For more specific bacterial species targeting, novel CRISPR-Cas systems and strategies may also be explored [99]. Regulatory concerns, including safety, measures for human applications, misuse risk assessment or dual-use implications, necessitate rigorous testing protocols with transparent communication channels, ethical guidelines pushed forth by international collaboration and oversight [99], strict regulations put in place [100], comprehensive risk assessments conducted [101]. Economic factors such as high-cost complexity for research & development associated with antimicrobials market entry rewards, extended patent protection grants, funding incentives [[102], [103], [104]] should all come into play as well. Additionally, resources need to be allocated equitably across low-income countries so that they can contribute meaningfully towards tackling this global issue. Collaboration and data sharing are integral to combating antimicrobial resistance but raise several challenges. To address these issues, interdisciplinary research networks and data repositories should be established [105] with collaborative licensing agreements and open access policies in place [106]. Furthermore, public awareness is essential to successfully adopting CRISPR-Cas technologies; thus, educational initiatives and transparent public engagement through outreach programs should be implemented while considering ethical implications [107]. Through such measures as mentioned in Table 5, we can move closer to addressing antimicrobial resistance with CRISPR-Cas technology.

**Table 5:** Challenges and opportunities in CRISPR-Cas applications against antimicrobial resistance, covering technical, regulatory, ethical, economic, and collaborative aspects.

Challenge Category	Specific Challenge	Opportunities and Solutions	References
Technical	Delivery of CRISPR-Cas components to target cells	Development of novel delivery methods, e.g., nanoparticles	[97]
	Off-target effects and unintended consequences	Improved specificity and design of CRISPR-Cas systems	[108]
	Rapid emergence of resistance to CRISPR-Cas therapies	Combinatorial therapies, continuous updating of targeting sequences	[98]
	Difficulty in targeting certain bacterial species	Exploration of novel CRISPR-Cas systems and targeting strategies	[109]
Regulatory and Ethical	Safety concerns regarding human and environmental applications	Rigorous testing, transparent communication, and ethical guidelines	[99]
	Potential for misuse or dual-use concerns	International collaboration and oversight, strict regulations	[100]
	Ethical implications of altering microbial ecosystems	Comprehensive risk assessment, ecological impact evaluation	[101]
Economic and Funding	Market and financial challenges for antimicrobial development	Market entry rewards, extended patent protection	[102]
	Limited resources for low-income countries	Global partnerships, allocation of resources and funding	[104]
	High cost and complexity of CRISPR-Cas research and development	Public-private partnerships, research grants, and funding incentives	[103]
Collaboration and Data Sharing	Intellectual property issues and licensing	Collaborative licensing agreements, open access policies	[106]
	Need for cross-disciplinary collaboration and data sharing	Establishment of interdisciplinary research networks and data repositories	[105]
Public Awareness and Education	Limited public understanding of CRISPR-Cas and antimicrobial resistance	Educational initiatives, public engagement, and outreach	[110]
	Public mistrust in genetic engineering and its applications	Transparency, public dialogue, and ethical considerations	[107]

## 9 Future perspectives

To guarantee a healthier future for all moving forward, we must blend CRISPR-Cas with emerging technological tools like artificial intelligence (AI) and machine learning (ML) [111]. By integrating the approaches, we can improve our odds of swiftly developing novel antimicrobial solutions. These technologies can enhance the precision and efficiency of CRISPR applications by optimizing guide RNA design, improving target specificity, and minimizing off-target effects. AI and ML can expedite the analysis and interpretation of extensive genetic datasets, accelerating the development of novel antimicrobial strategies and enabling personalized treatments based on individual genetic profiles. However, this integration also necessitates careful ethical considerations. Issues such as genetic data privacy [112,113], informed consent for genetic modifications [114,115], and the potential for algorithmic biases must be rigorously addressed [[116], [117], [118]].

In addition, recent advancements such as seekRNA and bridgeRNA introduce programmable RNA-guided DNA recombination systems that bypass the need for double-strand breaks, a typical limitation in CRISPR-based technologies [119,120]. These systems, derived from bacterial insertion sequences, employ single RNA molecules (seekRNA or bridgeRNA) to guide a recombinase to specific DNA sites, enabling precise genetic rearrangement, insertion, and excision [119,120]. SeekRNA and bridgeRNA can be reprogrammed to target specific genomic sites, providing a versatile platform for large-scale DNA manipulations with high specificity and minimal errors. This could have profound implications for AMR by allowing targeted excision or replacement of resistance genes, while their precision minimizes the risk of off-target effects, enhancing safety in complex microbial and human systems.

Advanced CRISPR-based gene replacement strategies, such as homology-directed repair (HDR), non-homologous end joining (NHEJ), and PASTE (Programmable Addition via Site-specific Targeting Elements), also hold promise for addressing AMR at the genomic level [[121], [122], [123]]. HDR utilizes a repair template to replace resistance genes with susceptible variants, though it remains technically challenging in bacteria [122]. NHEJ does not rely on a repair template; instead, it repairs DNA double-strand breaks by directly joining the broken DNA ends. This method is more error-prone and typically introduces small insertions or deletions at the break site. In the context of AMR, NHEJ can be used to disrupt resistance genes rather than replace them [123]. PASTE, combining CRISPR-Cas9 with integrase enzymes, enables site-specific DNA insertion, which could theoretically be used to remove or inactivate resistance genes directly [121].

Beyond these methods, other complex technologies offer innovative options for bacterial genome editing. Recombineering via RedET, for example, leverages homologous recombination to introduce large genetic sequences, potentially allowing for targeted replacement of entire resistance-related clusters in bacterial genomes [124]. Furthermore, CRISPR-associated transposon systems (CAST) offer a novel approach, as they integrate genes without relying on DNA repair pathways. CAST could be particularly valuable in bacterial applications, as it enables precise insertion of resistance-susceptibility genes without introducing double-strand breaks, a limitation in traditional CRISPR methods [125].

In agriculture and aquaculture, CRISPR-Cas could be employed to limit the use of antibiotics by developing disease-resistant crops and livestock [126,127]. Future studies should investigate CRISPR's impact on microbial communities and the environment, ensuring ecological safety. Regulatory and ethical considerations will be crucial for clinical, agricultural, and ecological applications of CRISPR, requiring policymakers and scientists to collaborate on global standards to manage these technologies responsibly.

Although there is currently limited data, CRISPR-Cas14a, a highly compact protein capable of cleaving ssDNA, holds significant potential for combating AMR. Its ability to target ssDNA without sequence restriction makes it an ideal tool for disrupting mobile genetic elements, such as plasmids, that often carry AMR genes. By cleaving ssDNA intermediates during plasmid replication or horizontal gene transfer, Cas14a could effectively prevent the spread of resistance genes. Additionally, its sequence-independent activity allows broad-spectrum targeting across diverse bacterial species, offering a promising avenue for future AMR interventions.

## 10 Conclusion

CRISPR-Cas technologies have emerged as pivotal tools in combating antimicrobial resistance (AMR), offering precise, adaptable interventions that may revolutionize traditional antimicrobial strategies. This review elucidates the efficacy of CRISPR-Cas in gene editing to directly target resistance genes, develop novel antimicrobial agents, and enhance diagnostics, potentially mitigating AMR impacts across clinical, agricultural, and environmental domains. Despite the promise, deploying CRISPR-Cas systems faces substantial challenges, including technical limitations such as off-target effects, complex delivery mechanisms, and robustness of gene editing outcomes. Furthermore, ethical concerns, regulatory compliance, and economic accessibility remain significant hurdles to their global application. Future research should prioritize refining CRISPR specificity and delivery techniques, establishing comprehensive regulatory standards, and fostering global cooperation to ensure equitable access to this technology. It is essential to address these issues to utilize CRISPR-Cas capabilities against AMR effectively. This has the potential to impact infectious disease management and public health greatly.

## CRediT authorship contribution statement

**Ahmed S.A. Ali Agha:** Writing – review & editing, Writing – original draft, Conceptualization. **Ali Al-Samydai:** Writing – review & editing. **Talal Aburjai:** Writing – original draft, Conceptualization.

## Ethics approval and consent to participate

Not applicable. This article contains no studies performed by authors with human participants or animals. It is a comprehensive review, synthesizing insights from previously published articles.

## Data availability statement

No data was used for the research described in the article.

## Declaration

We confirm that this manuscript is not under consideration elsewhere and that all authors have consented to its submission.

## Declaration of competing interest

The authors declare that they have no known competing financial interests or personal relationships that could have appeared to influence the work reported in this paper.
